# Transcriptome analysis of a wild bird reveals physiological responses to the urban environment

**DOI:** 10.1038/srep44180

**Published:** 2017-03-14

**Authors:** Hannah Watson, Elin Videvall, Martin N. Andersson, Caroline Isaksson

**Affiliations:** 1 Department of Biology, Lund University, SE-223 62 Lund, Sweden

## Abstract

Identifying the molecular basis of environmentally induced phenotypic variation presents exciting opportunities for furthering our understanding of how ecological processes and the environment can shape the phenotype. Urban and rural environments present free-living organisms with different challenges and opportunities, which have marked consequences for the phenotype, yet little is known about responses at the molecular level. We characterised transcriptomes from an urban and a rural population of great tits *Parus major*, demonstrating striking differences in gene expression profiles in both blood and liver tissues. Differentially expressed genes had functions related to immune and inflammatory responses, detoxification, protection against oxidative stress, lipid metabolism, and regulation of gene expression. Many genes linked to stress responses were expressed at higher levels in the urban birds, in accordance with our prediction that urban animals are exposed to greater environmental stress. This is one of the first studies to reveal transcriptional differences between urban- and rural-dwelling animals and suggests an important role for epigenetics in mediating environmentally induced physiological variation. The study provides valuable resources for developing further in-depth studies of the mechanisms driving phenotypic variation in the urban context at larger spatial and temporal scales.

Unravelling the mechanistic basis of how phenotypic traits respond – adaptively or non-adaptively - to novel or changing environments has major implications for our understanding and management of anthropogenic-induced environmental change. The world is currently witnessing unprecedented rates of urban development and urban areas represent the fastest-growing form of land cover^1^. While the urban environment may remove many of the ecological constraints that exist elsewhere, for example by providing year-round high food availability and reduced climatic stress in winters, towns and cities present free-living organisms with a wider range of environmental stressors, arising from air-, artificial night light-, water-, and noise-pollution, as well as changes in host-pathogen dynamics, compared with natural environments^2^,^3^,^4^,^5^. Despite the challenges posed by urban environments, many animals persist, colonise or even thrive in towns and cities, while others avoid urban areas entirely^2^. Marked changes in behaviour, physiology and morphology have all been widely demonstrated among organisms inhabiting urban areas, and diverse studies have contributed to our understanding of how ecological processes shape the phenotype in the urban environment^6^,^7^,^8^,^9^,^10^,^11^. However, despite this surge in interest in the ecological consequences of urbanisation, our understanding of the molecular basis of observed phenotypic variation is limited^12^.

Whole-genome quantification of gene expression, using high-throughput sequencing technologies, can be used to identify genes that contribute towards phenotypic variation between individuals and populations. Such studies have revealed genes and pathways that play important roles in mechanistic responses to environmental challenges in free-living organisms, for example the effects of ocean acidification in corals^13^, pollution tolerance in killifish^14^, and the molecular response to malaria infection^15^ and differences in gene expression along an altitudinal gradient^16^ in birds. Exposure to particulate matter from urban and rural environments induced changes in gene expression in laboratory-reared zebrafish embryos^17^, while a comparative study of humans resident in Flanders (Belgium) revealed correlations between expression levels of candidate genes and environmental carcinogens^18^. Harris *et al*.^19^ provided evidence of directional selection in transcriptomes of urban-dwelling wild mice, though the authors acknowledged the limitations of polymorphism data generated from pooled transcriptomes. In another study, transcriptomes from urban and rural mice were compared, but the study was designed to develop genomic resources and lacks sufficient controls to derive urban/rural comparisons^20^. Understanding the contribution of gene expression to environmentally induced phenotypic variation at the individual level in the urban environment will provide crucial insight into the regulation of the phenotype at the molecular level in response to urban stressors in the wild.

The great tit *Parus major* L. is a passerine bird that is common throughout Europe and widespread in both urban and rural habitats; hence, it has become a model system in avian urban ecology^6^,^21^,^22^. To gain insight into the key molecular mechanisms underlying physiological responses to urban environments, we performed high-throughput Illumina RNA sequencing (RNA-seq) to characterise replicated transcriptomes from liver and whole blood tissues from a pair of urban and rural great tit populations in Sweden. Both the avian liver and blood are sensitive to changes in diet^21^,^23^ and pollutant exposure^24^,^25^ and, therefore, are expected to contribute to differences in gene expression in relation to urbanisation^26^,^27^. The circulatory system also provides defence against antigens, such as particulate matter and pathogens, which may be important in underlying phenotypic differences between urban and rural animals^28^,^29^. Firstly, we quantified differences in gene expression between the urban and rural birds. Secondly, we performed gene enrichment analyses, using gene ontology (GO) annotation, to determine the biological processes and molecular functions that were overrepresented among differentially expressed genes. Our results revealed that the primary functional differences between urban and rural birds relate to immune and inflammatory responses, protection against oxidative stress, lipid metabolism, and regulation of gene expression. Furthermore, many genes linked to stress responses were expressed at higher levels in the urban-, compared with the rural-, dwelling birds; this suggests that differences in diet and stress exposure, e.g. pollution and pathogens, between the two environments may underlie the observed variation in gene expression. Whether our results, derived from a single pair of populations, reflect a general difference between urban and rural populations across larger spatial scales should be verified, and this study provides valuable resources to inform the design of future studies.

## Results and Discussion

### Urban and rural birds exhibit different gene expression profiles

**Figure 1:**
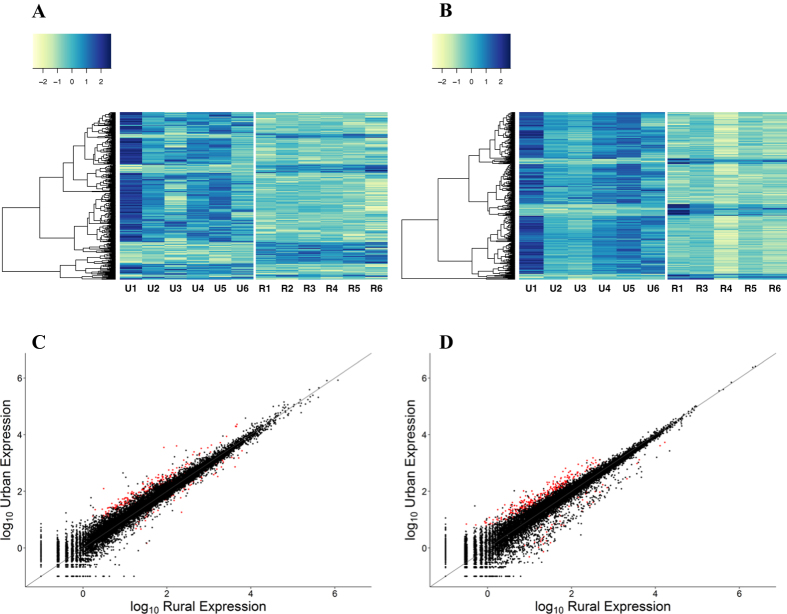
Differential gene expression between urban and rural great tits *Parus major*. Heatmaps illustrate expression levels of differentially expressed genes (rows) for each individual (columns) in **(A)** liver (n = 304 genes) and **(B)** whole blood (n = 372 genes). Individuals are denoted U1-U6 and R1-R6 for urban and rural individuals, respectively. Normalised gene expression has been variance-stabilised, and scaled and centred around zero to produce Z-scores. A darker colour indicates higher expression and a lighter colour indicates lower expression. Scatterplots show mean normalised gene expression levels (log_10_-transformed) in **(C)** liver (n = 12 individuals) and **(D)** blood (n = 11 individuals). Red points indicate significant differentially expressed genes (*q* < 0.1), whereas black points indicate genes that are not significantly differentially expressed. The blood transcriptome from individual R2 was removed from the analysis since it was identified as an outlier (see Supplementary Fig. S4).

Differential gene expression analyses, together with a principal components analysis (PCA; Supplementary Fig. S1), revealed that the urban- and rural-dwelling great tits exhibited significantly different expression profiles (Fig. 1; Supplementary Data S2 and S3). This pattern was evident in both liver and blood tissues: 304 and 372 genes were significantly differentially expressed (*q* < 0.1) between the two environments in liver and blood, respectively (Fig. 1; Supplementary Data S3). Supplementary Tables S1 and S2 show the top 20 most significant annotated genes that were differentially expressed in each of liver and blood tissues. Of those genes that were differentially expressed, the majority were expressed at higher levels in the urban (liver: 238 [78%]; blood: 321 [86%]), relative to the rural, environment. Neither the heatmaps (Fig. 1) nor PCA (Supplementary Fig. S1) revealed high similarities among the two different age classes (see Methods), and so we have no reason to believe that differences in age are driving the observed variation in gene expression between urban and rural individuals. Gene enrichment analysis revealed significant overrepresentation of 318 and 281 GO terms assigned to the differentially expressed genes in liver and blood transcriptomes, respectively (Supplementary Data S4). While only 9% (n = 53) of the differentially expressed genes were common to liver and blood (Supplementary Fig. S2A), 28% (n = 130) of the significantly overrepresented GO terms were common to both tissues (Supplementary Fig. S2B). This result indicates that although some of the environmentally induced functional responses operate in both liver and blood tissue, they largely involve different genes. Differences in expression profiles are expected, given the different functions of the two tissues, and emphasise the value of investigating transcriptional responses in other tissues besides blood. Many of the genes that were more highly expressed in urban, compared with rural, birds are involved in physiological and biochemical responses to the biotic and abiotic stressors that are characteristic of urban environments. In the proceeding sections, we evaluate and discuss the functional significance of the observed variation in gene expression. It should be noted that gene expression at the RNA level might not always correlate to actual protein levels^30^, though several studies have shown good correlation between mRNA and protein concentrations^31^,^32^.

### Urban upregulation of adaptive immune responses

**Figure 2:**
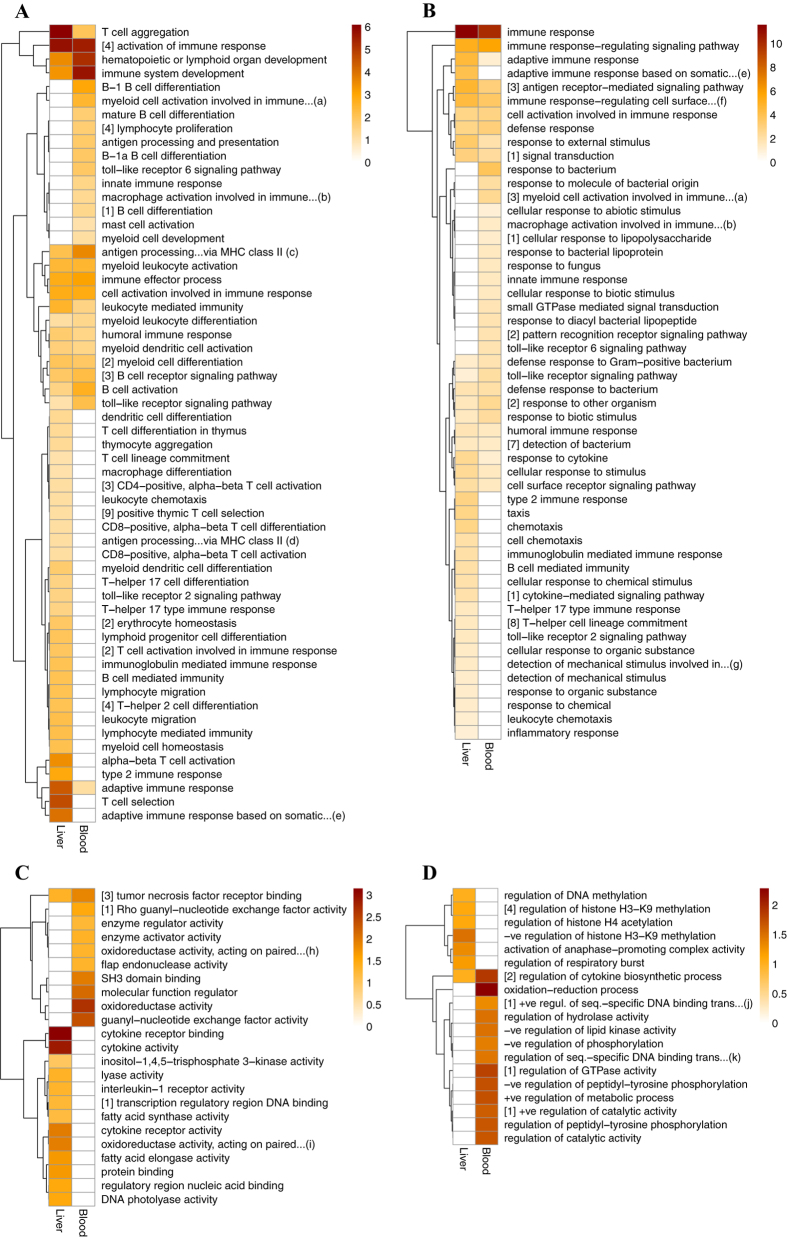
Key functional differences between urban and rural great tits *Parus major* in liver and blood tissues as revealed by gene enrichment analysis. Heatmaps indicate the significance level of overrepresentation of gene ontology (GO) terms nested within the parent terms: (**A**) ‘immune system process’, (**B**) ‘response to stimulus’; (**C**) ‘metabolic process’ and ‘regulation of metabolic process’; and, (**D**) ‘molecular function’. The colour scale represents significance levels (−log_10_-transformed *q*-values); a darker colour indicates greater statistical significance (i.e. lower *q*-value). Some significant GO terms are nested under similar functions to avoid redundancy; the number of hidden sub-terms is displayed in square brackets. Refer to methods for full details. Abbreviated terms: (a) myeloid cell activation involved in immune response; (b) macrophage activation involved in immune response; (c) antigen processing and presentation of peptide or polysaccharide antigen via MHC class II; (d) antigen processing and presentation of peptide antigen via MHC class II; (e) adaptive immune response based on somatic recombination of immune receptors built from immunoglobulin superfamily domains; (f) immune response-regulating cell surface receptor signaling pathway; (g) detection of mechanical stimulus involved in sensory perception; (h) oxidoreductase activity, acting on paired donors, with oxidation of a pair of donors resulting in the reduction of molecular oxygen to two molecules of water; (i) oxidoreductase activity, acting on paired donors, with incorporation or reduction of molecular oxygen, reduced flavin or flavoprotein as one donor, and incorporation of one atom of oxygen; (j) positive regulation of sequence-specific DNA binding transcription factor activity; (k) regulation of sequence-specific DNA binding transcription factor activity; −ve = negative; +ve = positive.

Genes involved in the development, activation and regulation of adaptive immune responses were significantly overrepresented among the differentially expressed genes from both liver and blood (Fig. 2A,B; Supplementary Data S4). Indeed, many of the most significant differentially expressed genes are involved in functions related to adaptive or innate immunity (Supplementary Tables S1 and S2). Overrepresented functions primarily relate to activation and differentiation of T-cells (antigen receptors) and B-cells (involved in antibody production; Fig. 2A), antigen presentation and processing (e.g. via dendritic cells and MHC; Fig. 2A), and their relevant signalling pathways (Fig. 2A,B). T-cells and B-cells are the key mediators of adaptive immunity, and when presented with an antigen or a signal from microbes, proliferation and differentiation of lymphocytes proceeds^33^. Remarkably, nearly all genes associated with the GO terms ‘immune system process’ (liver: n = 53/53; blood: n = 57/59) and ‘immune response’ (liver: n = 29/29; blood: 29/30) were expressed at higher levels in the urban, relative to the rural, birds (Supplementary Data S3). Urban birds also demonstrated significantly higher expression of numerous genes whose products are involved in the detection of, and responses to, bacteria and compounds originating from bacteria (Fig. 2B). These differences were more evident from blood (14 genes associated with 13 significant GO terms), compared with liver (6 genes associated with 3 significant GO terms), transcriptomes (Supplementary Data S3 and S4). Since the circulatory system provides a fundamental role in the body’s defence against bacterial invasion, environmentally induced changes in bacterial defences would be expected to be more pronounced in the blood, compared with other tissues.

Consistent higher expression of genes linked to immune functions in urban birds could be explained by higher pathogen diversity and/or prevalence - and thus increased frequency of exposure to microbes and antigens - in the urban, compared with the rural, environment. It has been shown that urban environments can influence host-pathogen interactions, via changes in prevalence, routes of transmission and susceptibility of wild animals to disease^29^,^34^. Yet, while some studies report higher rates of disease and parasitism in urban, compared with rural, bird populations^5^,^35^, others report reduced pathogen prevalence and intensity in urban birds^4^. If pathogen diversity and/or prevalence are indeed higher in urban environments, increased activation of immune responses may increase the ability of urban great tits to cope with urban-associated changes in host-pathogen dynamics. Indeed, a previous comparative analysis of 39 phylogenetically-independent events of urbanisation suggested that species that showed stronger immune responses (based on the size of the bursa of Fabricius) were more successful in invading urban areas than species with weaker immune responses^36^. However, enhanced immune activity may not necessarily mean birds can avoid the expected negative impacts on survival or reproduction associated with higher pathogen diversity and prevalence, since there may also be fitness costs associated with an upregulation of immune defences^37^. Regardless of the mechanisms involved, the transcriptome data from our urban and rural study populations suggest that characteristics of the adaptive immune system could be central to the underlying differences between urban- and rural-dwelling great tits.

### Urban birds have enhanced innate immune activity

Among the genes that were significantly differentially expressed between the urban and rural birds, GO terms linked to inflammatory response pathways were significantly overrepresented in both liver and blood transcriptomes (Fig. 2B–D). More specifically, overrepresented processes primarily relate to the production, secretion and receptor-binding of cytokines, and chemotaxis (liver: n = 41 genes associated with 29 significant GO terms; blood: n = 24 genes associated with 6 significant GO terms; Supplementary Data S3). Cytokines (e.g. tumour necrosis factors, chemokines, and interleukins) play an important role in host response to infection and trauma, by mediating inflammatory responses^33^,^38^ typified by redness, swelling, heat and pain^39^. Similar to our findings concerning the adaptive immune system, all but one of the genes associated with overrepresented inflammatory processes were expressed at higher levels in urban, relative to rural, birds (Supplementary Data S2 and S3). Increased expression of cytokine-encoding genes has been widely demonstrated in response to bacterial^40^, viral^41^ and parasitic^42^ infections in avian macrophage cells. The *CP* gene, coding for ceruloplasmin, is just one example of an inflammatory-linked gene that is expressed at higher levels in the urban birds; as well as playing a key role in iron detoxification (see below), ceruloplasmin is an acute phase protein that is synthesised in the liver during the acute phase of an inflammatory response and, as such, is an indicator of acute inflammation in birds^43^. Markers of inflammation are widely used in toxicological and biomedical studies, and enhanced inflammatory responses in humans have, in addition to being linked with infection and trauma, been associated with exposure to pollutants, such as heavy metals^44^, particulate matter^45^ and nitrogen oxides^46^. The observed differences in gene expression linked to innate immune activity between the urban and rural birds could therefore be explained by differences in exposure to pathogens, traffic-generated particulate matter, noise and/or artificial night light; increased exposure to all of these sources of environmental stress are likely to cause upregulation of inflammatory responses^12^,^47^.

While inflammatory responses are part of the normal innate immune response, excessive or uncontrolled inflammation contributes to tissue damage and disease^38^. Inflammation triggers an increased production of reactive oxygen species (ROS), which, if not effectively removed by antioxidant defences, leads to oxidative stress and damage to cellular macromolecules such as nucleic acids, lipids and proteins^48^. Thus, inflammation and oxidative stress are tightly linked. Increased oxidative stress is widely implicated in senescent declines in organismal function and the pathology of many cancers and cardiovascular diseases^48^. Although we have some understanding of how oxidative stress is affected by urban pollution, we know little, if anything, about inflammatory responses to urban stressors in wild animals^12^. While enhanced inflammation could be beneficial, providing defences to infection, injury and pollution associated with urban environments, persistent high activity of innate immune responses could also be degenerative for urban birds.

### Urban birds have more active detoxification and repair systems

Although the GO term ‘metal ion binding’ was not significantly overrepresented, genes associated with this term were numerous among the differentially expressed genes in both liver (n = 38) and blood (n = 63; Supplementary Data S3). Furthermore, the majority of genes were expressed at higher levels in the urban, compared with the rural, birds (liver: n = 23/38; blood: n = 54/63). Two genes coding for metallothioneins (*B5G2T6_TAEGU* and *MT4*) were not only expressed at significantly higher levels in the livers of urban, relative to rural, great tits, but were among the most significant of all the differentially expressed genes from liver transcriptomes (Supplementary Table S1). Urban great tits also exhibited significantly higher expression, in blood tissue, of a metalloprotein gene (*CP*) and three genes coding for metallopeptidases (*MME*, *MMP28*, *ADAM19*; Supplementary Data S3); the primary biological functions of all of the encoded proteins concern metal ion binding and metal detoxification.

Although metals are essential micronutrients for organisms, playing key roles in metabolic processes and signalling pathways, they can become toxic at higher concentrations^25^, partly as a consequence of oxidative stress^49^. The environmental toxicological literature abounds with studies demonstrating oxidative stress induced by exposure to pollutants in both terrestrial and aquatic environments^45^,^50^. While there is evidence for pollutant-induced oxidative stress in urban birds, our knowledge concerning the impacts and capacity for modulating detoxification pathways in birds is limited^28^,^51^. Our results suggest that the expression of genes involved in metal detoxification pathways could be important in underlying the functional differences between urban and rural birds. Metallothioneins (MT) play an important role in the detoxification of heavy metals^52^ and have been shown to be efficient scavengers of hydroxyl-radicals *in vitro*^53^. Concentrations of MT in liver and kidney have been shown to strongly correlate with levels of cadmium in great tits^24^, while exposure to a number of different metal ions has been shown to result in enhanced activity of various antioxidant enzymes and proteins, including MT, in fish^54^. Similarly, in mice, MT gene expression is upregulated in response to oxidative-stress-inducing agents, including metal ions, xenobiotics, and inflammation, as well as oxidative stress itself^52^,^55^. The observed high expression of genes involved in metal detoxification pathways in urban birds, in the present study, could represent a response to limit the damaging effects of elevated levels of pro-oxidants and reduce the toxic effects arising from exposure to heavy metals in the urban environment.

Among the genes that were significantly differentially expressed between urban and rural birds, there was significant overrepresentation of genes involved in oxidation-reduction activity (i.e. associated with the GO term ‘oxidoreductase activity’ and all directly descending terms) in both blood (n = 30; Fig. 2C) and liver (n = 3; Fig. 2C) tissues. For example, the urban birds exhibited higher expression of the *TXNRD1* gene, coding for thioredoxin reductase 1, responsible for the catalysis of the reduction of thioredoxin, which is an important cellular antioxidant. This suggests that the urban birds may have higher circulating levels of thioredoxin, which could be a response – either adaptive or non-adaptive - to exposure to high levels of oxidative stress. In addition, blood transcriptomes of the urban great tits revealed significantly higher expression of two genes encoding nucleases (*EXO1* and *FEN1*) that play key roles in DNA repair^56^. This result provides a further indication that urban birds could be exposed to higher levels of oxidation-induced DNA damage. A recent study from the same populations confirms this finding, by experimentally demonstrating that being reared in the urban environment leads to shorter telomeres^57^. Short telomeres have been widely associated with reduced lifespan and fitness, and oxidative stress is likely a key mediator of accelerated telomere attrition^58^. Taken together, our results suggest that differences in expression levels of genes related to mechanisms involved in detoxification and repair of oxidative damage correspond with the likely differences in exposure to oxidation-inducing chemicals between urban and rural individuals.

### Differential regulation of fatty acid physiology

Several genes that were significantly differentially expressed between the urban and rural birds are involved in lipid metabolism, binding and storage in both liver and blood (Supplementary Data S3). In particular, liver transcriptomes revealed significant overrepresentation of GO terms linked to the synthesis and elongation of fatty acids (n = 2 genes associated with 6 significant GO terms; Fig. 2C; Supplementary Data S4). The fatty acid composition of tissues is partly determined by the diet (which is likely to differ between urban and rural habitats), and has important implications for many physiological functions and processes including thermoregulation^59^, inflammation and oxidative stress^39^. These processes themselves are sensitive to extrinsic stressors, such as pollutants, associated with the urban environment^28^,^45^,^50^.

The rural great tits exhibited significantly higher expression, in the liver, of enzymes - two elongases (*ELOVL2*, *ELOVL5*) and a desaturase (*FADS6*) – involved in the biosynthesis of long-chain polyunsaturated fatty acids^60^,^61^ (PUFAs; Supplementary Data S3). Long-chain PUFAs are prone to lipid peroxidation^62^, thus having higher levels increases an organism’s susceptibility to oxidative damage upon exposure to pro-oxidants. As discussed above, our transcriptome data – in line with other studies – suggest that urban birds already face a greater oxidative threat; maintaining low expression of these elongases in the urban environment could therefore be a response to reduce the extent of oxidative damage to membrane lipids.

PUFAs are converted - via elongation and desaturation pathways - to eicosanoids^62^, which modulate the intensity and duration of inflammatory responses^39^. Typically, eicosanoids derived from the omega(ɷ)-6 PUFA pathway have pro-inflammatory effects (e.g. fever, vasodilation, oedema), while those derived from ɷ-3 PUFAs, have anti-inflammatory effects^39^. Although inflammatory responses act to combat harmful stimuli, excessive and uncontrolled inflammation can incur costs as a result of the generation of pro-oxidants and subsequent damage to macromolecules^48^. Interestingly, it was recently shown in our study populations that urban great tits had higher circulating levels of the ɷ-6 PUFA arachidonic acid, while rural great tits had higher levels of the ɷ-3 PUFA eicosapentaenoic acid^21^, suggesting that urban birds have higher levels of pro-inflammatory eicosanoid precursors. Although the relative preferences of the two elongases (*ELOVL2* and *ELOVL5*) for substrates from the ɷ-6 and ɷ-3 pathways^61^ have not been determined in birds (for mammalian studies, see 60,61), reducing expression of the genes in the urban environment may be a response to limit endogenous synthesis of pro-inflammatory PUFAs and a further increase in inflammation and subsequent oxidative damage.

### Epigenetic mechanisms as a possible mediator of responses to urban stressors

Expression profiles from liver and blood tissues revealed significant overrepresentation of several GO terms linked to the regulation of gene expression, via epigenetic modifications such as DNA methylation and histone methylation/acetylation, and via regulation of transcription factors (Fig. 2D; Supplementary Data S4). Furthermore, the specific genes concerned (liver: n = 15 genes; blood: n = 11 genes; Supplementary Data S3) are linked to DNA damage repair, tumour suppression, senescence and metal-ion binding, all of which relate to other observed transcriptional differences between the urban and rural great tits. Epigenetic mechanisms act to dynamically regulate the organisation and function of the genome, by regulating transcription and thus gene expression^63^. One of the most significant differentially expressed genes from liver transcriptomes encodes a methyltransferase (*MTR*; Supplementary Table S1), which catalyses the addition of methyl groups to DNA. DNA methylation is the most well-understood epigenetic mark and plays a crucial role in regulating gene expression. Furthermore, *MTR* was expressed at higher levels in rural, compared with urban, birds, suggesting that DNA-methylation levels of rural birds could be higher than those of urban birds in the study populations. Since methylation is typically associated with reduced gene expression^63^,^64^, higher expression of this methyltransferase gene in rural birds could contribute to overall lower levels of gene expression observed among the significant differentially expressed genes in these individuals (Fig. 1).

The results indicate a potential key role for epigenetic mechanisms in mediating the observed variation in gene expression, and likely subsequent phenotypic variation, between urban and rural birds. Indeed, recent sequencing of the great tit methylome indicated an important role for methylation in evolutionary processes within the species^65^, while methylation has also been shown to be important in shaping great tit personality^66^. The epigenome is highly responsive to environmental cues and aberrant patterns of DNA methylation have been linked with exposure to a wide range of environmental contaminants in humans and other animals^64^,^67^,^68^, as well as cardiovascular diseases, cancers and ageing^69^,^70^ in humans. Studies on laboratory rodents and humans have shown that patterns of methylation are particularly sensitive to early-life nutrition and may underpin disease susceptibility in later life^70^,^71^. More recently, diet was also shown to influence whole-genome methylation in wild baboons (*Papio cynocephalus*)^72^. The inferred differences in levels of DNA methylation between the urban and rural birds could therefore be a result of differences in pollutant exposure and/or diet between the two different environments. Importantly, since epigenetic modifications can be inherited during mitotic cell division, environmentally induced changes in gene expression can persist throughout life, resulting in life-long changes in phenotypic traits. Very little is known about epigenetic mechanisms in birds^73^, how patterns of methylation in birds are influenced by the environment, and whether epigenetic modifications are primarily the result of “developmental programming”.

### Conclusions

This is one of the first studies to quantify variation in genome-wide gene expression of a wild animal in the context of urbanisation. The existence of consistent and marked differences in gene expression profiles between urban and rural great tits, in the present study, suggests the regulation of responses at the molecular level to local environmental conditions. While the present study is limited by considering only a single pair of populations, many of the upregulated genes and functional differences were in the predicted direction and in accordance with previous physiological studies. For example, many genes linked to stress responses were expressed at higher levels in urban, compared with rural, birds, suggesting that the observed differences in gene expression are a result of differential stress-exposure in the two contrasting environments. The results suggest that differences in diet and exposure to pollutants and pathogens could all contribute to the observed variation in transcriptomes between urban and rural great tits. Future experimental studies should aim to determine the specific role of these different stressors and associated fitness consequences.

By offering novel insight into the biochemical and physiological functions involved in mediating responses to urban environmental stressors, our findings will facilitate identification of key regulatory genes in both liver and blood tissues. Since current sequencing costs restrict the viability of performing replicated transcriptome analyses across multiple pairs of urban/rural populations (while also retaining sufficient replication at the individual and tissue level), our results should be used to inform the application of candidate gene approaches to verify the consistency of the observed responses across broader spatial and temporal scales. It remains to be understood to what extent the observed transcriptomic differences between the urban and rural birds are the result of genetic adaptation, epigenetic modifications, and/or simply direct responses to ambient stressors. Our results, however, indicate that epigenetic mechanisms could play a key role in mediating the environmentally induced variation in gene expression in the context of urbanisation. Importantly, epigenetic mechanisms could generate life-long changes in gene expression and subsequently phenotypic traits.

## Methods

### Ethics statement

This study was carried out in accordance with Swedish legislation and approved by the Malmö-Lund animal ethics committee (Dnr M454 12:1).

### Study species and sites

Twelve wild male great tits – six from an urban environment and six from a rural environment - were euthanised in late winter 2014 in southern Sweden. Urban birds (denoted as U1-U6) were captured in Malmö (55°35′N 12°59′E; Supplementary Fig. S3A) - the third largest city in Sweden with c. 300,000 inhabitants. The study site in Malmö is an urban park that is characterised by a mix of coniferous and deciduous trees, managed grassland, ponds and associated urban infrastructure including paved footpaths and buildings. Rural birds (denoted as R1-R6) were captured in Vombs fure (55°39′N 13°33′E; Supplementary Fig. S3B), a rural site located 35 km northeast of Malmö. Vombs fure comprises ~12 km^2^ of, primarily coniferous, forest interspersed with broadleaved trees; the surrounding area is sparsely inhabited by humans (<5 inhabitants km^−2^). For further details of study sites, refer to^21^. Rural birds were captured seven days after urban birds. Since breeding occurs up to 7 days earlier in Malmö, compared with Vomb, we do not expect there to be differences between urban and rural birds with respect to physiological changes in anticipation of the breeding season. Birds were captured while roosting in nestboxes at night and transported to the laboratory in Lund where biometrics were recorded, and a blood sample was collected and immediately transferred to storage at −80 °C. Post-mortems were carried out immediately after sacrifice; liver tissue was collected and transferred to storage at −80 °C within 5 min of death. Rural birds (mean ± SE: 19.5 ± 0.25 g) were significantly heavier than urban birds (mean ± SE: 17.8 ± 0.22 g; T-test: t = 3.46, df = 6.8, *P* = 0.011), though there were no differences in body size, as measured by tarsus length (mean ± SE: rural: 22.9 ± 0.20, urban: 22.6 ± 0.79; T-test: t = 0.35, df = 4.5, *P* = 0.739). All birds were adults: rural birds were all aged 3 K + (i.e. 3 yr or older); the urban birds U1–3 were 2 K (i.e. 2nd calendar year), while U4-6 were 3 K+.

### RNA isolation, library preparation and sequencing

Total RNA was isolated from 25 mg liver tissue and 15 μl of whole blood for each of the 12 individuals. Samples were first homogenised in 60 μl (liver)/20 μl (blood) Autoclaved Millipore water using a TissueTearor (BioSpec, Bartlesville, USA). A single Trizol/chloroform extraction step was performed, followed by purification using Qiagen’s RNeasy kit (Hilden, Germany), according to the manufacturer’s instructions. RNA yield and purity were checked using a NanoDrop spectrophotometer (Thermo Scientific, USA) and an Agilent 2100 Bioanalyser (Santa Clara, USA). All samples were confirmed to be of acceptable quality as indicated by the 260/280 nm absorption ratio (median ± SD: 2.02 ± 0.05) and RIN (RNA Integrity Number; median ± SD: 7.1 ± 1.1). DNase treatment was performed prior to sequencing. Truseq library construction and 100 bp paired-end RNA-seq of individual samples was carried out using Illumina HiSeq 2000 by BGI Tech Solutions (Hong Kong). All samples were sequenced twice, in different lanes, and a mix of urban and rural samples were run in each lane. For reasons beyond our control, the RNA from the blood of R2 was sequenced separately from all other blood RNA samples; since the resulting transcriptome was highly dissimilar to all other transcriptomes (Supplementary Fig. 4), this individual was removed from the analysis of blood transcriptomes.

### Transcriptome analysis

Raw RNA-seq reads were multiplexed, and subjected to adaptor trimming and quality screening. We obtained 763 million and 772 million high-quality paired-end reads, from liver and blood tissues, respectively. Reads (1.5 billion; 767 million pairs) were mapped using TopHat2 2.0.12^74^ to the zebra finch *Taeniopygia guttata* genome (v.3.2.4) downloaded from Ensembl (www.ensembl.org). Although the great tit genome has recently been published^65^, within the passerines, the zebra finch has undoubtedly the highest-quality genome assembly, with 18,618 annotated genes compared with 13,036 genes in the great tit genome. Furthermore, GO information exists for 14,601 genes in the zebra finch genome, while GO data does not yet exist for the great tit. Avian genomes are highly conserved between species, making the use of a well-annotated genome from another species possible^75^. A maximum mismatch rate of 25% was allowed to account for species divergence. A total of 582 million (76.3%) and 632 million (81.9%) reads, from liver and blood tissues respectively, successfully aligned to the genome. Unique reads that mapped unambiguously to genes were counted using HTSeq 0.5.3p9^76^ and SAMtools 0.1.19^77^. All raw reads have been deposited in NCBI’s Sequence Read Archive (PRJNA314210).

### Differential gene expression analysis

Differential gene expression analyses were performed using DESeq2 1.6.3^78^. As per Love *et al*.^78^, individual counts were normalised using the geometric mean and modelled with a negative binomial distribution. Shrinkage of dispersion and fold-change estimates are implemented in DESeq2 to improve stability and interpretability, meaning that logarithmic fold-changes will have more shrinkage when there is little information available for a gene (i.e. low read count, high dispersion, or few degrees of freedom). *P*-values were corrected for false positives using the Benjamini and Hochberg false discovery rate (FDR) correction^79^ for multiple testing, within DESeq2. Genes with an FDR (or *q*-value) <0.1 were regarded as statistically significantly different. Hierarchical clustering, using Principal Component Analysis (PCA), was performed on expression data from liver and blood tissues (Supplementary Fig. S1A–C). Expression values were transformed using the recommended variance stabilizing transformation procedure for use in PCA and to generate heatmaps (Fig. 1A,B). One individual (R2) was removed from the analysis of blood transcriptomes, since the PCA and distance heatmaps revealed it to be an extreme outlier (Supplementary Fig. S4), possibly due to it being sequenced in a separate sequencing run.

### Gene enrichment analysis

Functional annotations and overrepresentation of GO terms were analysed using the Cytoscape plug-in BiNGO 3.0.2^80^. A full ontology file for the zebra finch was downloaded from www.geneontology.org (08-18-2015). Gene enrichment analyses were performed using a hypergeometric test for overrepresentation of GO terms from the differentially expressed gene set, using the zebra finch GO as the background. Again, *P*-values were corrected using the Benjamini and Hochberg FDR, within BiNGO, and GO terms with a *q*-value < 0.1 were regarded as statistically significantly different. We evaluated GO terms from the biological process and molecular function domains. For the production of heatmaps in Fig. 2, GO term sets were condensed using REVIGO^81^.

### Data accessibility:

RNA-seq raw reads are deposited in NCBI’s Sequence Read Archive (PRJNA314210).

## Additional Information

**How to cite this article:** Watson, H. *et al*. Transcriptome analysis of a wild bird reveals physiological responses to the urban environment. *Sci. Rep.* **7**, 44180; doi: 10.1038/srep44180 (2017).

**Publisher's note:** Springer Nature remains neutral with regard to jurisdictional claims in published maps and institutional affiliations.

## Supplementary Material

Supplementary Information

Supplementary Dataset 1

Supplementary Dataset 2

Supplementary Dataset 3

## References

[b1] Seto K. C., Guneralp B. & Hutyra L. R. Global forecasts of urban expansion to 2030 and direct impacts on biodiversity and carbon pools. Proc. Natl. Acad. Sci. 109, 16083–16088 (2012). 22988086 10.1073/pnas.1211658109 PMC3479537

[b2] McKinney M. L. Urbanization, biodiversity and conservation. Bioscience 52, 883–890 (2002).

[b3] Shochat E., Warren P. S., Faeth S. H., Mcintyre N. E. & Hope D. From patterns to emerging processes in mechanistic urban ecology. Trends Ecol. Evol. 21, 186–191 (2006). 16701084 10.1016/j.tree.2005.11.019

[b4] Evans K. L. . Effects of urbanisation on disease prevalence and age structure in blackbird *Turdus merula* populations. Oikos 118, 774–782 (2009).

[b5] Giraudeau M., Mousel M., Earl S. & McGraw K. Parasites in the city: degree of urbanization predicts poxvirus and coccidian infections in house finches (*Haemorhous mexicanus*). PLoS One 9, e86747 (2014). 24503816 10.1371/journal.pone.0086747 PMC3913573

[b6] Slabbekoorn H. & Peet M. Birds sing at a higher pitch in urban noise. Nature 424, 267 (2003). 10.1038/424267a 12867967

[b7] Isaksson C., Örnborg J., Stephensen E. & Andersson S. Plasma glutathione and carotenoid coloration as potential biomarkers of environmental stress in great tits. Ecohealth 2, 138–146 (2005).

[b8] Liker A., Papp Z., Bókony V. & Lendvai A. Z. Lean birds in the city: body size and condition of house sparrows along the urbanization gradient. J. Anim. Ecol. 77, 789–795 (2008). 18479344 10.1111/j.1365-2656.2008.01402.x

[b9] Cheptou P.-O., Carrue O., Rouifed S. & Cantarel A. Rapid evolution of seed dispersal in an urban environment in the weed *Crepis sancta*. Proc. Natl. Acad. Sci. USA 105, 3796–3799 (2008). 18316722 10.1073/pnas.0708446105 PMC2268839

[b10] Stone E. L., Jones G. & Harris S. Street Lighting Disturbs Commuting Bats. Curr. Biol. 19, 1123–1127 (2009). 19540116 10.1016/j.cub.2009.05.058

[b11] Tennessen J. B., Parks S. E. & Langkilde T. Traffic noise causes physiological stress and impairs breeding migration behaviour in frogs. Conserv. Physiol. 2, 1–8 (2014). 10.1093/conphys/cou032 PMC4806738 27293653

[b12] Isaksson C. Urbanization, oxidative stress and inflammation: a question of evolving, acclimatizing or coping with urban environmental stress. Funct. Ecol. 29, 913–923 (2015).

[b13] Moya A. . Whole transcriptome analysis of the coral *Acropora millepora* reveals complex responses to CO2-driven acidification during the initiation of calcification. Mol. Ecol. 21, 2440–2454 (2012). 22490231 10.1111/j.1365-294X.2012.05554.x

[b14] Whitehead A., Triant D. A., Champlin D. & Nacci D. Comparative transcriptomics implicates mechanisms of evolved pollution tolerance in a killifish population. Mol. Ecol. 19, 5186–5203 (2010). 20874759 10.1111/j.1365-294X.2010.04829.x

[b15] Videvall E., Cornwallis C. K., Palinauskas V., Valkiūnas G. & Hellgren O. The avian transcriptome response to malaria infection. Mol. Biol. Evol. 32, 1255–1267 (2015). 25636457 10.1093/molbev/msv016 PMC4408411

[b16] Cheviron Z. A., Whitehead A. & Brumfield R. T. Transcriptomic variation and plasticity in rufous-collared sparrows (*Zonotrichia capensis*) along an altitudinal gradient. Mol. Ecol. 17, 4556–4569 (2008). 18986500 10.1111/j.1365-294X.2008.03942.x

[b17] Mesquita S. R. . Differential embryotoxicity of the organic pollutants in rural and urban air particles. Environ. Pollut. 206, 535–542 (2015). 26298234 10.1016/j.envpol.2015.08.008

[b18] van Leeuwen D. M. . Transcriptome analysis in peripheral blood of humans exposed to environmental carcinogens: a promising new biomarker in environmental health studies. Environ. Health Perspect. 116, 1519–1525 (2008). 19057705 10.1289/ehp.11401 PMC2592272

[b19] Harris S. E., Munshi-South J., Obergfell C. & O’Neill R. Signatures of rapid evolution in urban and rural transcriptomes of white-footed mice (*Peromyscus leucopus*) in the New York Metropolitan Area. PLoS One 8, e74938 (2013). 24015321 10.1371/journal.pone.0074938 PMC3756007

[b20] Harris S. E., O’Neill R. J. & Munshi-South J. Transcriptome resources for the white-footed mouse (*Peromyscus leucopus*): new genomic tools for investigating ecologically divergent urban and rural populations. Mol. Ecol. Resour. 15, 382–394 (2015). 24980186 10.1111/1755-0998.12301 PMC4281516

[b21] Andersson M. N., Wang H.-L., Nord A., Salmón P. & Isaksson C. Composition of physiologically important fatty acids in great tits differs between urban and rural populations on a seasonal basis. Front. Ecol. Evol. 3, 93 (2015).

[b22] de Jong M. . Effects of nocturnal illumination on life-history decisions and fitness in two wild songbird species. Philos. Trans. R. Soc. Lond. B. Biol. Sci. 370, 1–8 (2015). 10.1098/rstb.2014.0128 PMC4375368 25780240

[b23] Møller A. P., Erritzøe J. & Karadas F. Levels of antioxidants in rural and urban birds and their consequences. Oecologia 163, 35–45 (2010). 20012100 10.1007/s00442-009-1525-4

[b24] Vanparys C. . Metallothioneins (MTs) and delta-aminolevulinic acid dehydratase (ALAd) as biomarkers of metal pollution in great tits (*Parus major*) along a pollution gradient. Sci. Total Environ. 401, 184–193 (2008). 18499231 10.1016/j.scitotenv.2008.04.009

[b25] Koivula M. J. & Eeva T. Metal-related oxidative stress in birds. Environ. Pollut. 158, 2359–2370 (2010). 20382455 10.1016/j.envpol.2010.03.013

[b26] Jaenisch R. & Bird A. Epigenetic regulation of gene expression: how the genome integrates intrinsic and environmental signals. Nat. Genet. 33 Suppl, 245–254 (2003). 12610534 10.1038/ng1089

[b27] Fisher M. A. & Oleksiak M. F. Convergence and divergence in gene expression among natural populations exposed to pollution. BMC Genomics 8, 108 (2007). 17459166 10.1186/1471-2164-8-108 PMC1868758

[b28] Isaksson C. Pollution and its impact on wild animals: a meta-analysis on oxidative stress. Ecohealth 7, 342–350 (2010). 20865439 10.1007/s10393-010-0345-7

[b29] Delgado-V. C. A. & French K. Parasite-bird interactions in urban areas: current evidence and emerging questions. Landsc. Urban Plan. 105, 5–14 (2012).

[b30] Mayfield A. B., Wang Y.-B., Chen C.-S., Chen S.-H. & Lin C.-Y. Dual-compartmental transcriptomic+ proteomic analysis of a marine endosymbiosis exposed to environmental change. Mol. Ecol. 2, 5944–5958 (2016). 10.1111/mec.13896 27778414

[b31] Lu P., Vogel C., Wang R., Yao X. & Marcotte E. M. Absolute protein expression profiling estimates the relative contributions of transcriptional and translational regulation. Nat. Biotechnol. 25, 117–124 (2007). 17187058 10.1038/nbt1270

[b32] Li J. J., Bickel P. J. & Biggin M. D. System wide analyses have underestimated protein abundances and the importance of transcription in mammals. PeerJ 2, e270 (2014). 24688849 10.7717/peerj.270 PMC3940484

[b33] Schat K. A., Kaspers B. & Kaiser P. Avian Immunology. (Elsevier Ltd 2013).

[b34] Bradley C. A. & Altizer S. Urbanization and the ecology of wildlife diseases. Trends Ecol. Evol. 22, 95–102 (2007). 17113678 10.1016/j.tree.2006.11.001 PMC7114918

[b35] Bradley C. A., Gibbs S. E. J. & Altizer S. Urban land use predicts West Nile virus exposure in songbirds. Ecol. Appl. 18, 1083–1092 (2008). 18686573 10.1890/07-0822.1

[b36] Møller A. P. Successful city dwellers: a comparative study of the ecological characteristics of urban birds in the Western Palearctic. Oecologia 159, 849–858 (2009). 19139922 10.1007/s00442-008-1259-8

[b37] Råberg L., Nilsson J.-A., Ilmonen P., Stjernman M. & Hasselquist D. The cost of an immune response: vaccination reduces parental effort. Ecol. Lett. 3, 382–386 (2000).

[b38] Libby P. Inflammatory mechanisms: the molecular basis of inflammation and disease. Nutr. Rev. 65, S140–S146 (2007). 18240538 10.1111/j.1753-4887.2007.tb00352.x

[b39] Calder P. C. Polyunsaturated fatty acids, inflamation and immunity. Eur. J. Clin. Nutr. 56, S14–S19 (2002). 12142955 10.1038/sj.ejcn.1601478

[b40] Wigley P. . Macrophages isolated from chickens genetically resistant or susceptible to systemic salmonellosis show magnitudinal and temporal differential expression of cytokines and chemokines following *Salmonella enterica* challenge. Infect. Immun. 74, 1425–1430 (2006). 16428798 10.1128/IAI.74.2.1425-1430.2006 PMC1360331

[b41] Palmquist J. M. . *In vivo* activation of chicken macrophages by infectious bursal disease virus. Viral Immunol. 19, 305–315 (2006). 16817773 10.1089/vim.2006.19.305

[b42] Dalloul R. A. . Unique responses of the avian macrophage to different species of Eimeria. Mol. Immunol. 44, 558–566 (2007). 16563507 10.1016/j.molimm.2006.02.004

[b43] Chamanza R. . Serum amyloid A and transferrin in chicken. A preliminary investigation of using acute-phase variables to assess diseases in chickens. Vet. Q. 21, 158–162 (1999). 10568007 10.1080/01652176.1999.9695012

[b44] Stohs S. J. & Bagchi D. Oxidative mechanisms in the toxicity of metal ions. Free Radic. Biol. Med. 18, 321–336 (1995). 7744317 10.1016/0891-5849(94)00159-h

[b45] Oberdörster G., Stone V. & Donaldson K. Toxicology of nanoparticles: a historical perspective. Nanotoxicology 1, 2–25 (2007).

[b46] Last J. A., Sun W. M. & Witschi H. Ozone, NO, and NO2: oxidant air pollutants and more. Environ. Health Perspect. 102, S179–S184 (1994). 10.1289/ehp.94102s10179 PMC1566979 7705295

[b47] Stroh E., Harrie L. & Gustafsson S. A study of spatial resolution in pollution exposure modelling. Int. J. Health Geogr. 6, 19 (2007). 17547740 10.1186/1476-072X-6-19 PMC1892775

[b48] Halliwell B. & Gutteridge J. M. C. Free Radicals in Biology and Medicine. (Oxford University Press, 2007).

[b49] Ercal N., Gurer-Orhan H. & Aykin-Burns N. Toxic metals and oxidative stress part I: mechanisms involved in metal induced oxidative damage. Curr. Top. Med. Chem. 1, 529–539 (2001). 11895129 10.2174/1568026013394831

[b50] Valavanidis A., Vlahogianni T., Dassenakis M. & Scoullos M. Molecular biomarkers of oxidative stress in aquatic organisms in relation to toxic environmental pollutants. Ecotoxicol. Environ. Saf. 64, 178–189 (2006). 16406578 10.1016/j.ecoenv.2005.03.013

[b51] Fossi M. C., Leonzio C., Focardi S., Lari L. & Renzoni A. Modulation of mixed-function oxidase activity in black-headed gulls living in anthropogenic environments: biochemical acclimatization or adaptation? Environ. Toxicol. Chem. 10, 1179–1188 (1991).

[b52] Andrews G. K. Regulation of metallothionein gene expression by oxidative stress and metal ions. Biochem. Pharmacol. 59, 95–104 (2000). 10605938 10.1016/s0006-2952(99)00301-9

[b53] Abel J. & de Ruiter N. Inhibition of hydroxyl-radical-generated DNA degradation by metallothionein. Toxicol. Lett. 47, 191–196 (1989). 2545017 10.1016/0378-4274(89)90075-1

[b54] Lushchak V. I. Environmentally induced oxidative stress in aquatic animals. Aquat. Toxicol. 101, 13–30 (2011). 21074869 10.1016/j.aquatox.2010.10.006

[b55] Bauman J. W., Liu J., Liu Y. P. & Klaassen C. D. Increase in metallothionein produced by chemicals that induce oxidative stress. Toxicol. Appl. Pharmacol. 110, 347–354 (1991). 1891778 10.1016/s0041-008x(05)80017-1

[b56] Matsuzaki Y., Adachi N. & Koyama H. Vertebrate cells lacking FEN-1 endonuclease are viable but hypersensitive to methylating agents and H2O2. Nucleic Acids Res. 30, 3273–3277 (2002). 12136109 10.1093/nar/gkf440 PMC135760

[b57] Salmon P., Nilsson J. F., Nord A., Bensch S. & Isaksson C. Urban environment shortens telomere length in nestling great tits, *Parus major*. Biol. Lett. 12, 8–11 (2016). 10.1098/rsbl.2016.0155 PMC4938044 27303051

[b58] Monaghan P. & Haussmann M. F. Do telomere dynamics link lifestyle and lifespan? Trends Ecol. Evol. 21, 47–53 (2006). 16701469 10.1016/j.tree.2005.11.007

[b59] Ben-Hamo M., McCue M. D., McWilliams S. R. & Pinshow B. Dietary fatty acid composition influences tissue lipid profiles and regulation of body temperature in Japanese quail. J. Comp. Physiol. B Biochem. Syst. Environ. Physiol. 181, 807–816 (2011). 10.1007/s00360-011-0558-2 21328066

[b60] Leonard A. E. . Identification and expression of mammalian long-chain PUFA elongation enzymes. Lipids 37, 733–740 (2002). 12371743 10.1007/s11745-002-0955-6

[b61] Inagaki K. . Identification and expression of a rat fatty acid elongase involved in the biosynthesis of C18 fatty acids. Biosci. Biotechnol. Biochem. 66, 613–621 (2002). 12005057 10.1271/bbb.66.613

[b62] Larsson S. C., Kumlin M., Ingelman-Sundberg M. & Wolk A. Dietary long-chain n-3 fatty acids for the prevention of cancer: a review of potential mechanisms. Am. J. Clin. Nutr. 79, 935–945 (2004). 15159222 10.1093/ajcn/79.6.935

[b63] Goldberg A. D., Allis C. D. & Bernstein E. Epigenetics: a landscape takes shape. Cell 128, 635–638 (2007). 17320500 10.1016/j.cell.2007.02.006

[b64] Head J. A. Patterns of DNA methylation in animals: an ecotoxicological perspective. Integr. Comp. Biol. 54, 77–86 (2014). 24785828 10.1093/icb/icu025

[b65] Laine V. N. . Evolutionary signals of selection on cognition from the great tit genome and methylome. Nat. Commun. 7, 10474 (2016). 26805030 10.1038/ncomms10474 PMC4737754

[b66] Verhulst E. C. . Evidence from pyrosequencing indicates that natural variation in animal personality is associated with DRD4 DNA methylation. Mol. Ecol. 25, 1801–1811 (2016). 26678756 10.1111/mec.13519

[b67] Baccarelli A. . Rapid DNA methylation changes after exposure to traffic particles. Am. J. Respir. Crit. Care Med. 179, 572–578 (2009). 19136372 10.1164/rccm.200807-1097OC PMC2720123

[b68] Baccarelli A. & Bollati V. Epigenetics and environmental chemicals. Curr. Opin. Pediatr. 21, 243–251 (2009). 19663042 10.1097/mop.0b013e32832925cc PMC3035853

[b69] Laird P. W. Cancer epigenetics. Hum. Mol. Genet. 14, R65–R76 (2005). 15809275 10.1093/hmg/ddi113

[b70] Gluckman P. D., Hanson M. A., Buklijas T., Low F. M. & Beedle A. S. Epigenetic mechanisms that underpin metabolic and cardiovascular diseases. Nat. Rev. Endocrinol. 5, 401–408 (2009). 19488075 10.1038/nrendo.2009.102

[b71] Dolinoy D. C., Weidman J. R. & Jirtle R. L. Epigenetic gene regulation: linking early developmental environment to adult disease. Reprod. Toxicol. 23, 297–307 (2007). 17046196 10.1016/j.reprotox.2006.08.012

[b72] Lea A. J., Altmann J., Alberts S. C. & Tung J. Resource base influences genome-wide DNA methylation levels in wild baboons (*Papio cynocephalus*). Mol. Ecol. 25, 1681–1696 (2016). 26508127 10.1111/mec.13436 PMC4846536

[b73] Frésard L. . Epigenetics and phenotypic variability: some interesting insights from birds. Genet. Sel. Evol. 45, 16 (2013). 23758635 10.1186/1297-9686-45-16 PMC3693910

[b74] Kim D. . TopHat2: accurate alignment of transcriptomes in the presence of insertions, deletions and gene fusions. Genome Biol. 14, R36 (2013). 23618408 10.1186/gb-2013-14-4-r36 PMC4053844

[b75] Ellegren H. Evolutionary stasis: the stable chromosomes of birds. Trends Ecol. Evol. 25, 283–91 (2010). 20363047 10.1016/j.tree.2009.12.004

[b76] Anders S., Pyl P. T. & Huber W. HTSeq—a Python framework to work with high-throughput sequencing data. Bioinformatics 31, 166–169 (2014). 25260700 10.1093/bioinformatics/btu638 PMC4287950

[b77] Li H. . The Sequence Alignment/Map format and SAMtools. Bioinformatics 25, 2078–2079 (2009). 19505943 10.1093/bioinformatics/btp352 PMC2723002

[b78] Love M. I., Huber W. & Anders S. Moderated estimation of fold change and dispersion for RNA-seq data with DESeq2. Genome Biol. 15, 550 (2014). 25516281 10.1186/s13059-014-0550-8 PMC4302049

[b79] Benjamini Y. & Hochberg Y. Controlling the false discovery rate: a practical and powerful approach to multiple testing. J. R. Stat. Soc. Ser. B 57, 289–300 (1995).

[b80] Maere S., Heymans K. & Kuiper M. BiNGO: a Cytoscape plugin to assess overrepresentation of gene ontology categories in biological networks. Bioinformatics 21, 3448–3449 (2005). 15972284 10.1093/bioinformatics/bti551

[b81] Supek F., Bošnjak M., Škunca N. & Šmuc T. Revigo summarizes and visualizes long lists of gene ontology terms. PLoS One 6, e21800 (2011). 21789182 10.1371/journal.pone.0021800 PMC3138752

