# Supplementary material for: Transcriptome analysis of a wild bird reveals physiological responses to the urban environment
Source: Sci Rep. 2017 Mar 14;7:44180. doi: 10.1038/srep44180 (PMC5349542; doi:10.1038/srep44180)

Supplementary Information

Appendix S1

Transcriptome analysis of a wild bird reveals physiological responses to the urban environment

Hannah Watson[1*], Elin Videvall[1], Martin N. Andersson[1] and Caroline Isaksson[1]

[1]Department of Biology, Lund University, SE-223 62 Lund, Sweden

*Corresponding author: hannah.watson@biol.lu.se

Contents

Supplementary Table S1. The top 20 most significant genes that were differentially expressed between urban (n = 6) and rural (n = 6) great tits *Parus major* from liver transcriptomes.

Supplementary Table S2. The top 20 most significant genes that were differentially expressed between urban (n = 6) and rural (n = 5) great tits *Parus major* from whole blood transcriptomes.

Supplementary Figure S1. Principal Components Analysis (PCA) plots illustrating clustering within habitats among transcriptomes from **(A)** liver and **(B)** whole blood from urban and rural great tits *Parus major*.

Supplementary Figure S2. Venn diagrams showing overlap of significant **(A)** differentially-expressed genes and **(B)** overrepresented gene ontology terms between whole blood (n = 11) and liver (n = 12) transcriptomes from urban and rural great tits *Parus major*.

Supplementary Figure S3. Aerial photographs indicating the location of sampling sites (yellow pins) and surrounding habitat for the **(A)** urban and **(B)** rural great tit *Parus major* study populations in southern Sweden.

Supplementary Figure S4. Plots illustrating the dissimilarity of an outlying sample (R2) from a transcriptome analysis of great tits *Parus major* from an urban (U1-U6) and a rural (R1-R6) environment.

Supplementary Table S1. The top 20 most significant annotated genes that were differentially expressed between urban (n = 6) and rural (n = 6) great tits *Parus major* from liver transcriptomes. Reads were mapped to the zebra finch *Taeniopygia guttata* genome. Gene names and descriptions are derived from UniProtKB. A positive log_2-fold change indicates higher expression in urban, relative to rural, birds and *vice-versa*.

**Gene ID**	**Gene name**	**Gene description**	***q*-value**	**Log_2 fold change**
ENSTGUG00000006533	B5G2T6_TAEGU	putative metallothionein II variant 2	2.15E-11	1.94
ENSTGUG00000010458	MTR	5-methyltetrahydrofolate-homocysteine methyltransferase	7.50E-11	-1.37
ENSTGUG00000006540	MT4	metallothionein 4	2.49E-07	1.97
ENSTGUG00000010883	IL22RA2	interleukin 22 receptor, alpha 2	4.02E-07	2.97
ENSTGUG00000001417	PTTG1	pituitary tumour-transforming 1	1.86E-05	2.08
ENSTGUG00000004224	CFHR5	complement factor H-related 5	5.58E-05	1.54
ENSTGUG00000005958	RRBP1	ribosome binding protein 1	2.44E-04	1.08
ENSTGUG00000015674	IL4I1	interleukin 4 induced 1	3.22E-04	2.01
ENSTGUG00000011171	SMC4	structural maintenance of chromosomes 4	3.22E-04	1.51
ENSTGUG00000008863	SAAL1	serum amyloid A-like 1	5.09E-04	1.85
ENSTGUG00000008410	AKAP13	A kinase (PRKA) anchor protein 13	6.93E-04	0.8
ENSTGUG00000002266	CSF3	colony stimulating factor 3 (granulocyte)	8.58E-04	2.2
ENSTGUG00000006329	MLKL	mixed lineage kinase domain-like	1.10E-03	1.6
ENSTGUG00000003999	ARHGAP25	Rho GTPase activating protein 25	1.59E-03	1.83
ENSTGUG00000009300	IL1RAP	interleukin 1 receptor accessory protein	1.60E-03	1.55
ENSTGUG00000000240	CD3D	CD3d molecule, delta (CD3-TCR complex)	1.67E-03	1.63
ENSTGUG00000012896	CSMD1	CUB and Sushi multiple domains 1	1.67E-03	-2.21
ENSTGUG00000010938	RPL22L1-1	uncharacterised gene	1.81E-03	1.49
ENSTGUG00000006488	PLA2R1	phospholipase A2 receptor 1, 180kDa	1.83E-03	1.08
ENSTGUG00000007022	COBLL1	cordon-bleu WH2 repeat protein-like 1	2.17E-03	-0.58

Supplementary Table S2. The top 20 most significant annotated genes that were differentially expressed between urban (n = 6) and rural (n = 5) great tits *Parus major* from whole blood transcriptomes. Reads were mapped to the genome of the zebra finch *Taeniopygia guttata* genome. Gene names and descriptions are derived from UniProtKB. A positive $\log_2$-fold change indicates higher expression in urban, relative to rural, birds and vice-versa.

Gene ID	Gene name	Gene description	*q*-value	Log_2 fold change
ENSTGUG00000001528	DOCK2	dedicator of cytokinesis 2	1.10E-08	2.03
ENSTGUG00000009502	PCTP	phosphatidylcholine transfer protein	1.10E-08	2.48
ENSTGUG00000003581	BANK1-2	uncharacterised gene	3.76E-08	2.48
ENSTGUG00000003282	CD79B	CD79b molecule, immunoglobulin-associated beta	4.27E-08	2.68
ENSTGUG00000000422	SYK	spleen tyrosine kinase	5.61E-08	2.34
ENSTGUG00000007659	PXYLP1	2-phosphoxylose phosphatase 1	1.98E-07	2.48
ENSTGUG00000004184	CDH23	cadherin-related 23	1.98E-07	2.75
ENSTGUG00000003230	SCN4A	sodium channel, voltage gated, type IV alpha subunit	2.04E-06	2.83
ENSTGUG00000008108	SWAP70	SWAP switching B-cell complex 70kDa subunit	4.51E-06	2.16
ENSTGUG00000002355	PARP8	poly (ADP-ribose) polymerase family, member 8	7.16E-06	1.97
ENSTGUG00000011987	LAPTM4B	lysosomal protein transmembrane 4 beta	1.47E-05	1.78
ENSTGUG00000000350	NFKBID	nuclear factor of kappa light polypeptide gene enhancer in B-cells inhibitor, delta	1.47E-05	2.61
ENSTGUG00000003579	BANK1-1	uncharacterised gene	1.47E-05	2.28
ENSTGUG00000011304	MME	membrane metallo-endopeptidase	2.35E-05	2.17
ENSTGUG00000008313	FAS	Fas cell surface death receptor	2.42E-05	2.35
ENSTGUG00000018506	GPR18	G protein-coupled receptor 18	2.42E-05	2.12
ENSTGUG00000011883	ARHGAP15	Rho GTPase activating protein 15	2.42E-05	1.92
ENSTGUG00000005185	SFRP2	secreted frizzled-related protein 2	2.42E-05	2.33
ENSTGUG00000007865	SLC6A15	solute carrier family 6 (neutral amino acid transporter), member 15	2.42E-05	1.82
ENSTGUG00000008023	CARD11	caspase recruitment domain family, member 11	7.13E-05	2.06

Supplementary Figure S1. Principal Components Analysis (PCA) plots illustrating clustering within habitats among transcriptomes from **(A)** liver and **(B)** whole blood from urban (blue; denoted U1-U6) and rural (red; denoted R1-R6, excluding R2) great tits *Parus major*. The variance associated with each of PC1 and PC2 is shown.

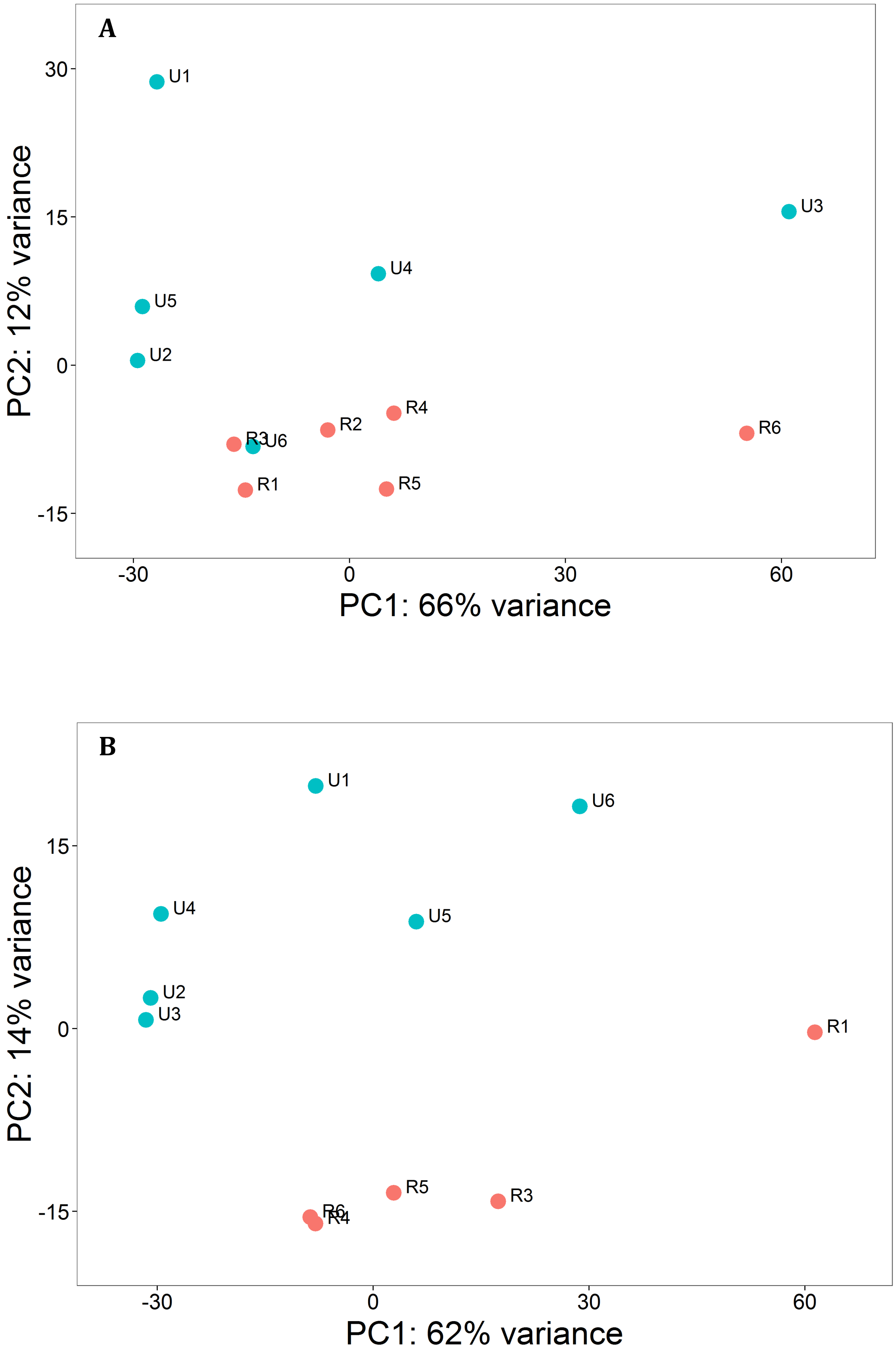

Supplementary Figure S2. Venn diagrams showing overlap of significant **(A)** differentially-expressed genes and **(B)** overrepresented gene ontology terms between whole blood (n = 11) and liver (n =12) transcriptomes from urban and rural great tits *Parus major*.

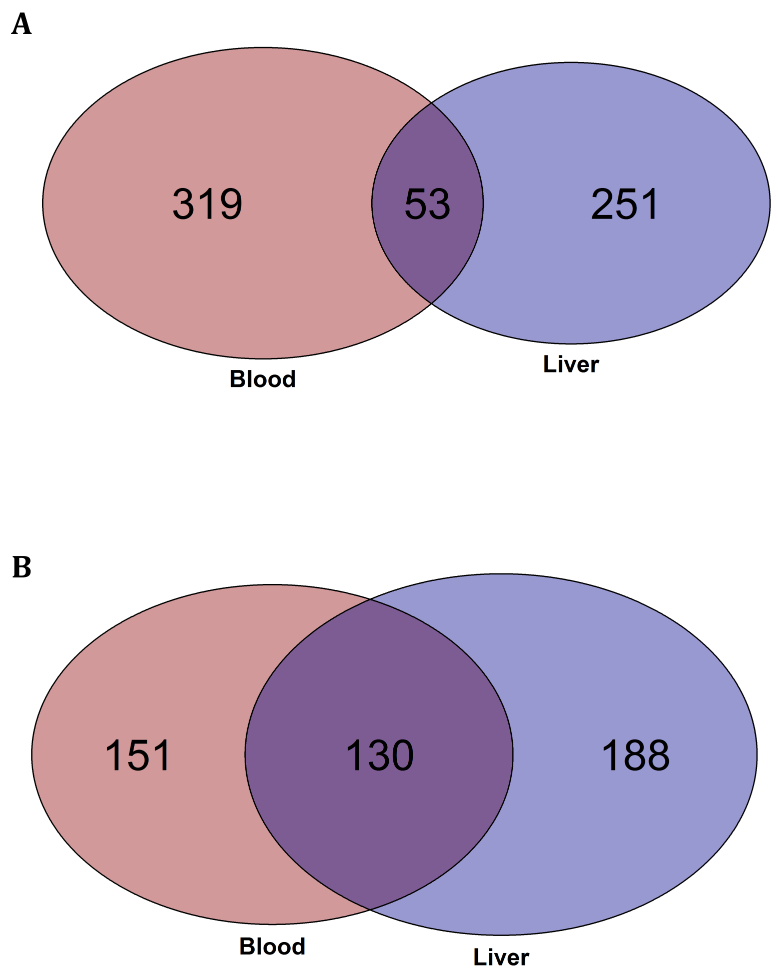

Supplementary Figure S3. Aerial photographs indicating the location of sampling sites (yellow pins) and surrounding habitat for the **(A)** urban and **(B)** rural great tit *Parus major* study populations in southern Sweden. The urban site is a city park located in Malmö (55°35'N 12°59'E) and the rural site is a forest in Vomb (55°39'N 13°33'E), which is located 35 km northeast of Malmö. The satellite images were taken from Google Earth (2015) and are reproduced at identical scales for direct comparison.

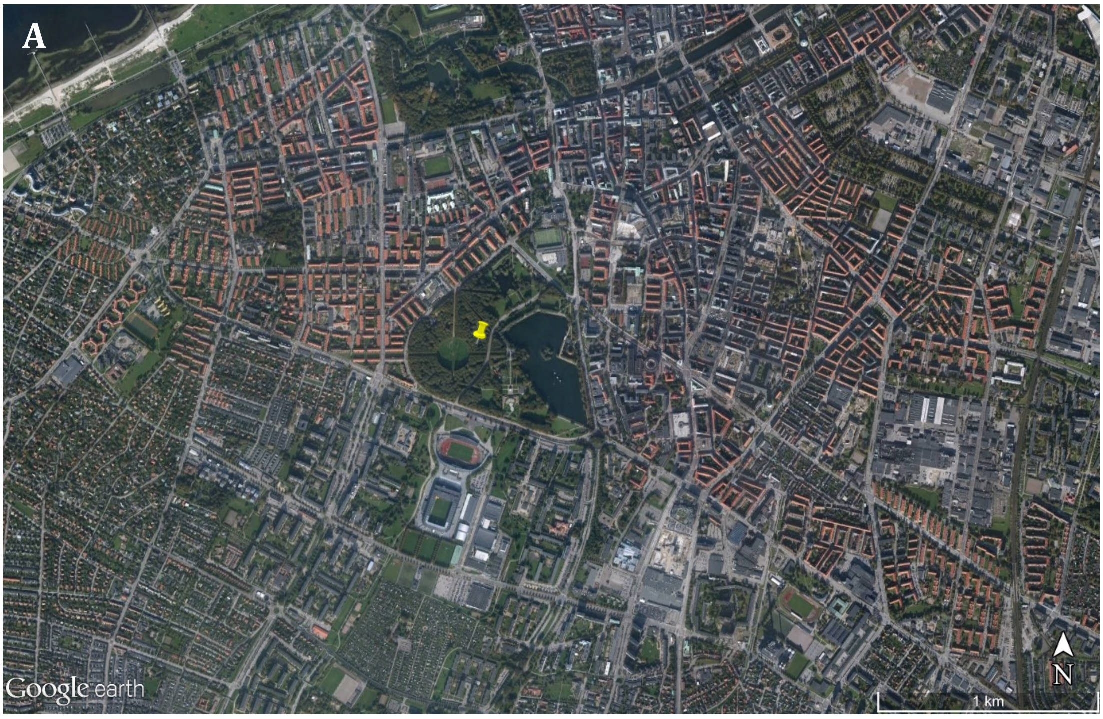

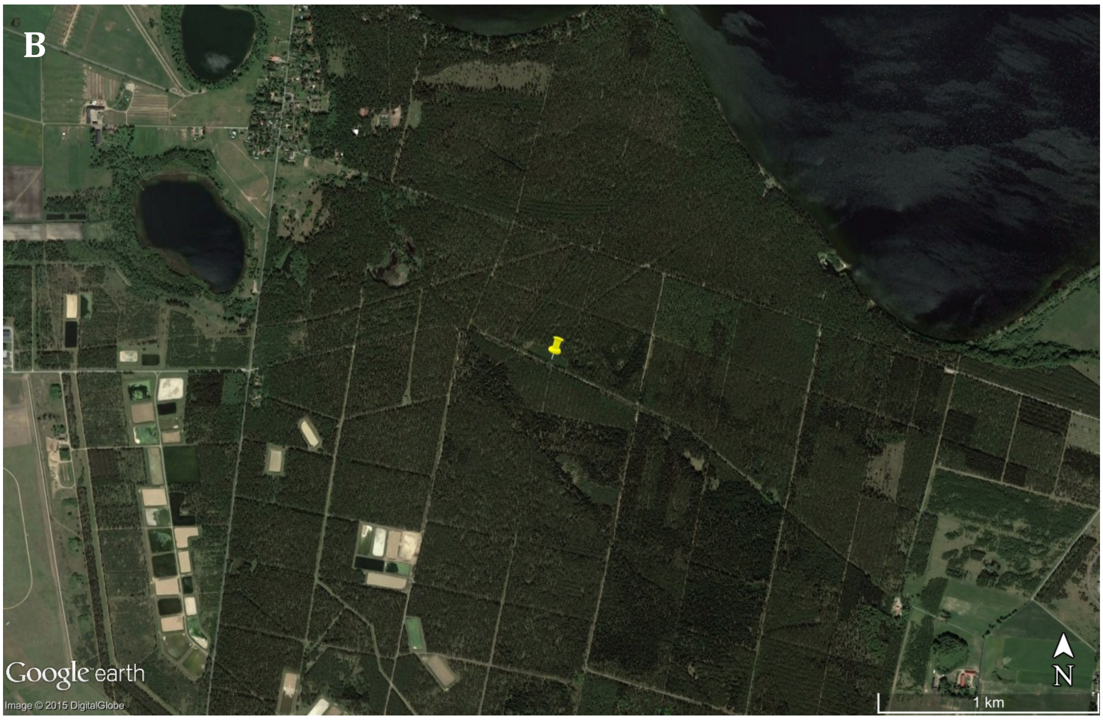

Supplementary Figure S4. Plots illustrating the dissimilarity of an outlying sample (R2) from a transcriptome analysis of great tits *Parus major* from an urban (U1-U6) and a rural (R1-R6) environment. The **(A)** scatterplot, generated from Principal Components 1 and 2, indicates the high dissimilarity of the blood transcriptome of R2 in relation to the transcriptomes of all other individuals; and, **(B)** heatmap illustrates Euclidean distance between the blood transcriptomes. A lighter colour indicates a shorter distance, while a darker colour indicates a greater distance to other transcriptomes. The variance associated with each of PC1 and PC2 is shown in (A). Due to the high dissimilarity of R2 to all other samples, it was removed from the main analyses of blood transcriptomes in the accompanying paper.

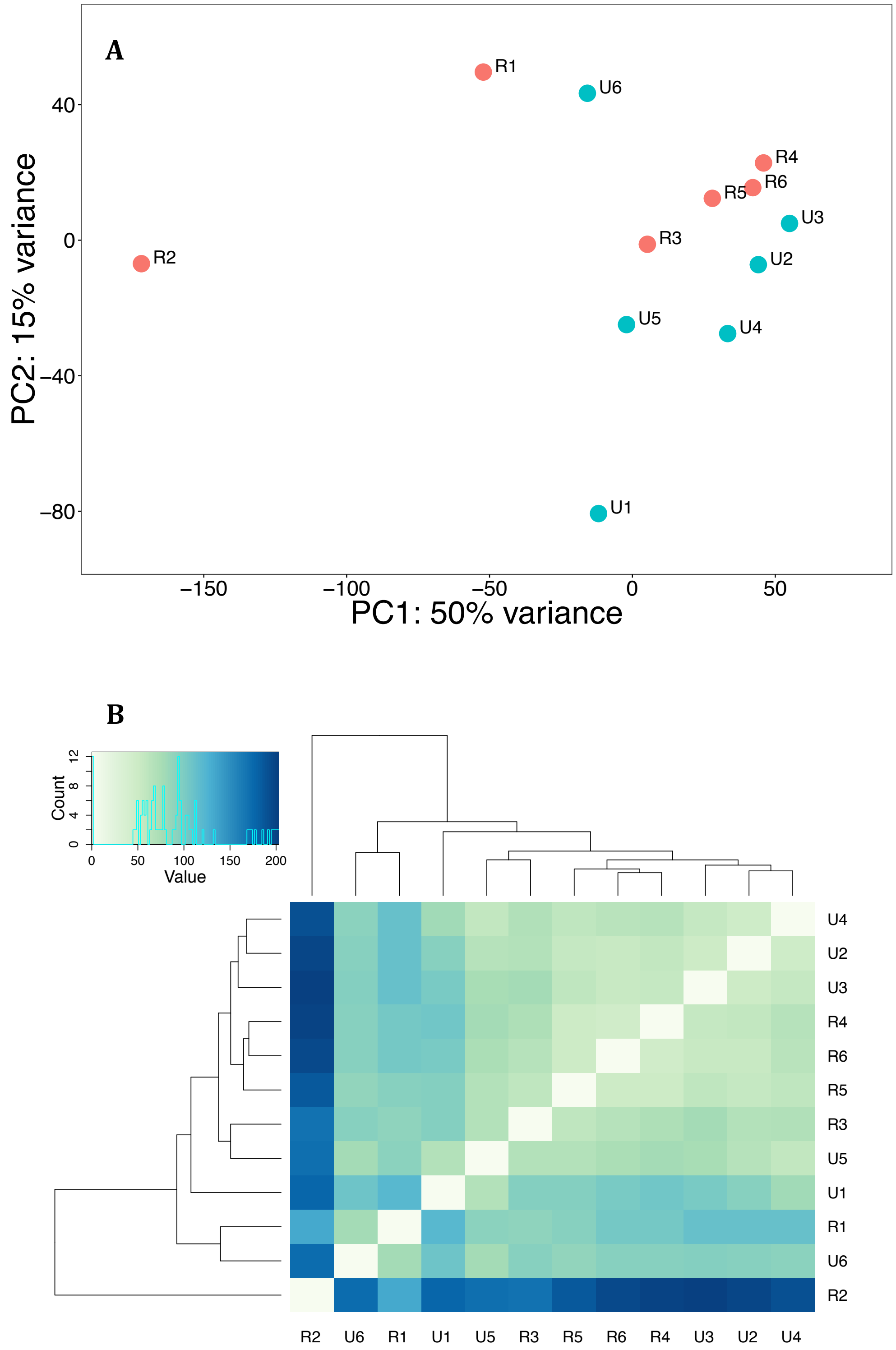

Supplement: Supplementary Information [file srep44180-s1.pdf]
